# The influence of polymorphic GSTM1 gene on the increased susceptibility of non-viral hepatic cirrhosis: evidence from observational studies

**DOI:** 10.1186/s40001-018-0331-z

**Published:** 2018-06-19

**Authors:** Ye Gu, Jing Zhao, Li Ao, Jianning Ma, Kena Bao, Min Liu, Weiping Huang

**Affiliations:** 1 Department of Nursing, Jiading District Central Hospital Affiliated Shanghai University of Medicine & Health Sciences, 1 Cheng Bei Road, Jiading District, Shanghai, 201800 China; 2 Department of Scientific Research, Jiading District Central Hospital Affiliated Shanghai University of Medicine & Health Sciences, 1 Cheng Bei Road, Jiading District, Shanghai, 201800 China

**Keywords:** Hepatic cirrhosis (HC), GSTM1, Polymorphism

## Abstract

It is reported that glutathione *S*-transferase mu (GSTM1) polymorphism is associated with non-viral hepatic cirrhosis (HC). However, some studies showed different views. Therefore, in this paper, a meta-analysis was conducted to get a more comprehensive understanding of GSTM1 polymorphisms in non-viral HC susceptibility. The results showed that GSTM1 null was associated with the increased risk of non-viral HC (OR = 1.337, 95% CI 1.112–1.804, *p* = 0.005). Subgroup analysis of cirrhosis type revealed that GSTM1 null was a prominent risk factor for alcoholic HC (OR = 1.416, 95% CI 1.112–1.804, *p* = 0.005). Meanwhile, subgroup analysis of population indicated that the significant differences only existed in Asian population (OR = 1.719, 95% CI 1.212–2.438, *p* = 0.002). In hospital-based studies, patients with GSTM1 null were more likely in risk of HC (OR = 1.426, 95% CI 1.092–1.863, *p* = 0.009). Subgroup analysis using genotyping method showed a significant association between GSTM1 null genotype and HC occurrence in the studies employing the multiple PCR genotyping method (OR = 1.559, 95% CI 1.171–2.076, *p* = 0.002). Based on the results of this analysis, it was concluded that GSTM1 null genotype could increase the susceptibility of non-viral hepatic cirrhosis. In addition, alcohol intake, Asian ethnicity, sample source from hospital and multiple PCR genotyping method may also influence the susceptibility of hepatic cirrhosis.

## Background

Hepatic cirrhosis (HC) expresses in dysfunction of liver due to normal liver tissue are gradually replaced by necrotic hepatocytes, which is caused by different etiology [1]. Patients with progressed HC suffer from a series of symptoms including diarrhea, ascites and esophageal variceal rupture bleeding [2]. As a chronic disease, HC brings a heavy burden to both the patients’ families and the society [3]. Cirrhosis affected 2.8 million people’s health and caused 1.3 million deaths in 2015 [4]. HC has become a worldwide public concern.

HC are mainly caused by hepatitis virus infection (hepatitis B, hepatitis C), heavy alcohol consumption and exposure to some chemical substances [5–7]. At present, viral hepatitis has been effectively prevented by health education and vaccine application. However, non-viral hepatic cirrhosis accounts for 51.4% of all cirrhosis, deserving more attention due to multiple influence factors. A number of studies have shown that genetic factors are responsible for the cirrhosis development other than environmental factors [8–10].

Glutathione *S*-transferase (GST) gene family is involved in the biotransformation phase II of harmful substances and has an important function to protect the cellular [11, 12]. The major GSTs family isoforms can be categorized into *α* (alpha), *μ* (mu), *θ* (theta), and *∏* (pi) classes [13]. Glutathione *S*-transferase mu (GSTM1) is one of the most widely expressed gene [14]. Null genotype was reported to be the most common variant of GSTM1 among populations [15]. It was reported that individuals with null genotype of the GSTM1 had no ability to detoxicate the xenobiotics [16]. Soto-Quintana et al. [17] reported that GSTM1 null genotype was related to a group of diseases including cancers and metabolic disorder, which may result in the vulnerability of liver tissue.

A lot of studies were performed to explore the association of GSTM1 gene polymorphism with cirrhosis risk. GSTM1 null genotype has been found to be related with non-viral hepatic cirrhosis in some studies [18–20]. However, several reports showed no significant correlation between GSTM1 null genotype and non-viral hepatic cirrhosis [21–23]. In this paper, a meta-analysis was performed to investigate the association between GSTM1 gene polymorphism and non-viral cirrhosis susceptibility.

## Methods

### Search strategy

Literatures were searched to find all the related articles in Pubmed, Web of Science, Embase databases (ultimate search updated on July 31, 2017) using the keywords “polymorphism”, “cirrhosis”, “chronic liver disease”, “glutathione *S*-transferase M1 (GSTM1)”. Two independent reviewers screened the relevant articles using standardized screening guide. The eligible articles were enrolled in this meta-analysis according to the inclusion and exclusion criteria.

### Inclusion and exclusion criteria

Studies meeting all of the following inclusion criteria are included: (a) included studies must be concentrated on the relationship between glutathione *S*-transferase M1 and the non-viral hepatic cirrhosis. (b) All enrolled studies must be the case–control studies. (c) Hepatic cirrhosis must be diagnosed on the basis of liver biopsy. (d) Published in English. (e) Studies with enough data to calculate odds ratios and corresponding 95% confidence intervals (ORs, 95% CIs) were included.

Exclusion criteria were as follows: (a) reviews, abstracts, letters, comments, family-based studies and single-case reports were excluded. (b)The articles with insufficient data or overlapped data were excluded.

### Quality assessment

We evaluate the quality of eligible studies with a modified 0–10 point scale, which is the appropriate quality assessment for case–control study [7, 24]. Quality evaluation parameters and standards of this modified scoring system (range 0–10 points) are shown in Table 1. The higher the article scores, the better quality the article has. The average score of the eligible studies is 7.33 points.

**Table 1:** Quality criteria for eligible studies

Quality parameters	Score		
	2	1	0
Population sample	> 100	50–100	< 50
Study design	Case and control group were both selected from hospital	Control groups were selected from normal residents	Unknown
General information^a^	Complete	Partial	Inadequate
Matching of case group and control group	> 3 factors	1–3 factors	None
Detection methods	Multiplex PCR	PCR–RFLP	Other methods

^a^Family history, medical history, life style habits and frequency of alcohol intake

### Data extraction

Two investigators (M. Liu and Y. Gu) extracted data independently. Any disagreement was settled by discussion. The extracted data included name of the first author, year of publication, country, ethnicity, number of cases and controls, genotyping method, control sources and genotype distribution in cases and controls.

### Statistical analysis

This meta-analysis was performed using STATA software (version12.0, STATA Corp, College Station, TX). Crude odds ratios (ORs) and corresponding 95% confidence intervals (CIs) were calculated to assess the strength of association between glutathione *S*-transferase M1 and the cirrhosis. Pooled ORs were calculated using random-effect model (M–H heterogeneity method) or fixed-effect model (Mantel and Haenszel method). *I*^2^ index and *p* value of the Chi-squared test were used to inspect the heterogeneity among the enrolled literature [25]. If notable heterogeneity existed (*p* < 0.05 and/or *I*^2^ > 50%), the random-effect model was used to estimate Ors [26], on the contrary, the fixed-effect model was performed [27]. Subgroup analysis was performed on cirrhosis type and population. The *Z* test and *p* value of 0.05 were used to judge whether the differences of OR values had statistical significance. Sensitivity analysis was conducted to assess the influence of individual studies. Begg’s test was applied to evaluate the publication bias [28].

## Results

### Search strategy and characteristics of eligible articles

**Fig. 1:**
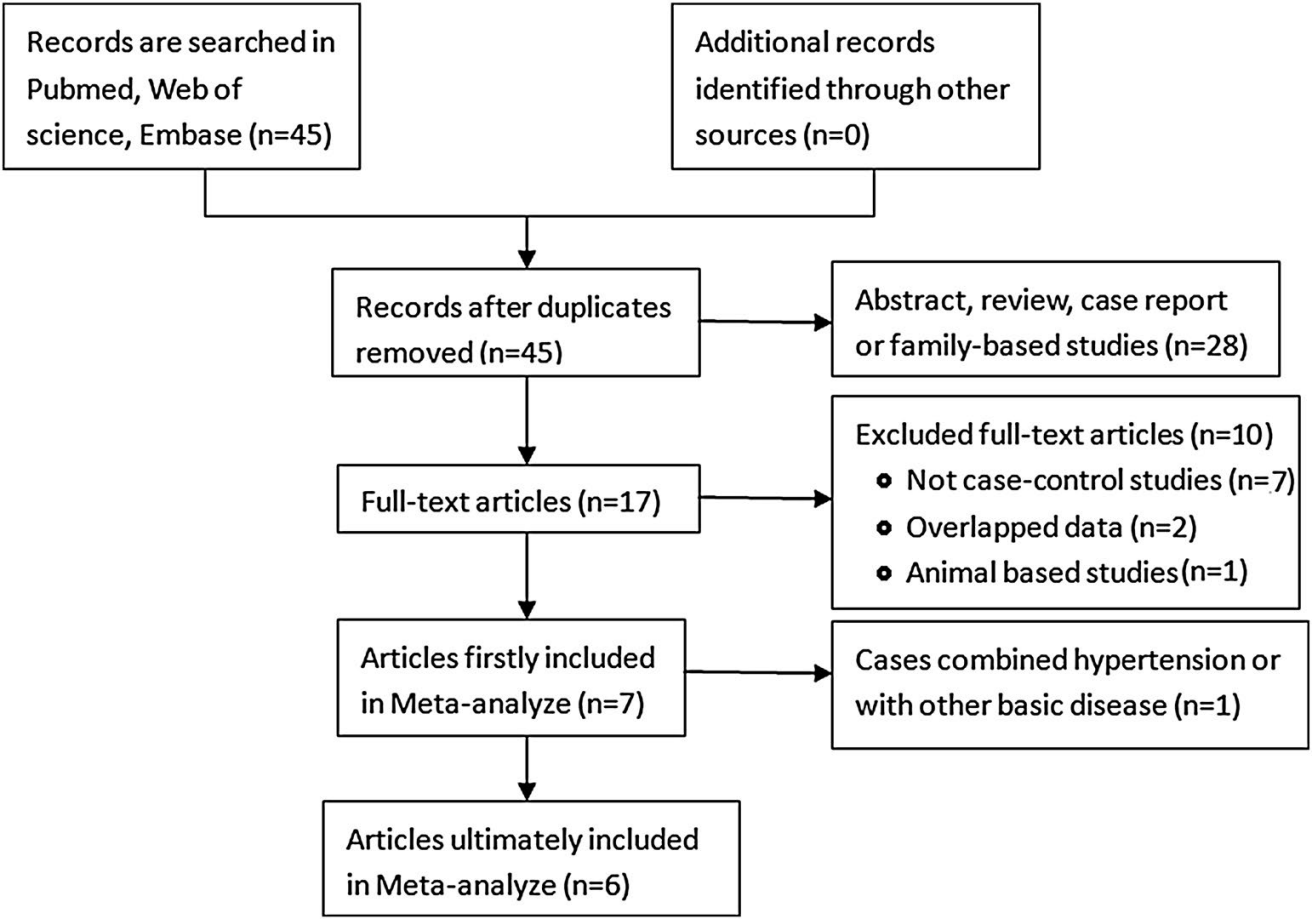
The flow chart of studies identification

The complete searching procedure is shown in Fig. 1. Six eligible studies were included in this meta-analysis on the basis of the inclusion and exclusion criteria. The general information of the eligible articles including the first author, publication year, original country, cirrhosis type, genotyping method, control source, and numbers of cases and controls were collected by two independent investigators. The characteristics of included studies and the distribution of genotype frequency of GSTM1 among controls and cirrhotic patients are shown in Table 2. 621 cases and 786 controls from these articles were employed. In addition, the total case numbers and control numbers of included studies were collected to calculate the pooled OR.

**Table 2:** Studies characteristics of each article included in the meta-analysis and distribution of genotype frequency of GSTM1 among controls and cirrhotic patients

First author	Year	Country	Cirrhosis type	Sample size (case/control)	Genotyping method	Control source	Matched factors	Score	Case group		Control group	
									GSTM1 active, *N* (%)	GSTM1 null, *N* (%)	GSTM1 active, *N* (%)	GSTM1 null, *N* (%)
Khan [18]	2010	India	Alcoholic HC	140/175	Multiple PCR	HB	Age, ethnicity, region	8	98 (56.0%)	77 (44.0%)	96 (68.5%)	44 (31.5%)
Khan [19]	2009	India	Alcoholic HC	160/100	Multiple PCR	HB	Region	8	92 (57.0%)	68 (43.0%)	70 (70.0%)	30 (30.0%)
Burim [21]	2004	Brazil	Alcoholic HC	65/221	Multiple PCR	HB	Age, gender, ethnicity, drink habits	9	35 (53.8%)	30 (46.2%)	120 (54.3%)	101 (45.8%)
Frenzer [20]	1999	Australia	Alcoholic HC	57/57	Multiple PCR	HB	Age, ethnicity, drink habits	8	3 (5.3%)	54 (94.7%)	10 (17.5%)	47 (82.5%)
Rodrigo [22]	2005	Spain	Alcoholic HC	120/200	PCR–RFLP	PB	Sex, region	6	51 (42.5%)	69 (57.5%)	90 (45.0%)	110 (55.0%)
Davies [23]	1993	Birmingham	Primary biliary HC	44/68	Horizontal starch gel electrophoresis	HB	None	5	27 (61.0%)	17 (39.0%)	37 (54.4%)	31 (45.6%)

*HB* hospital based, *PB* population based

### Results of meta-analysis

**Fig. 2:**
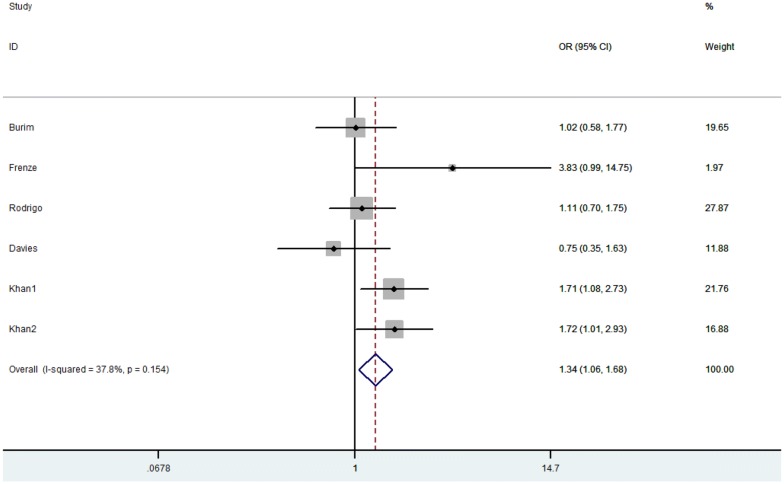
Forest plots of GSTM1 comparison (null vs. active) in all eligible articles

The between-study heterogeneity of all the five eligible studies was first analyzed and no significant heterogeneity was found (*p* = 0.154, *I*^2^ = 37.8%, Fig. 2). Thus, the fixed-effect model was used to assess the strength of the relationship between GSTM1 null genotype and risk of hepatic cirrhosis. Compared with control groups, the pooled OR of GSTM1 null in non-viral hepatic cirrhosis is 1.337 (95% CI 1.062–1.684, *p* = 0.013, Fig. 2), which indicates that null GSTM1 is associated with an increased risk of non-viral hepatic cirrhosis.

### Subgroup analysis

**Fig. 3:**
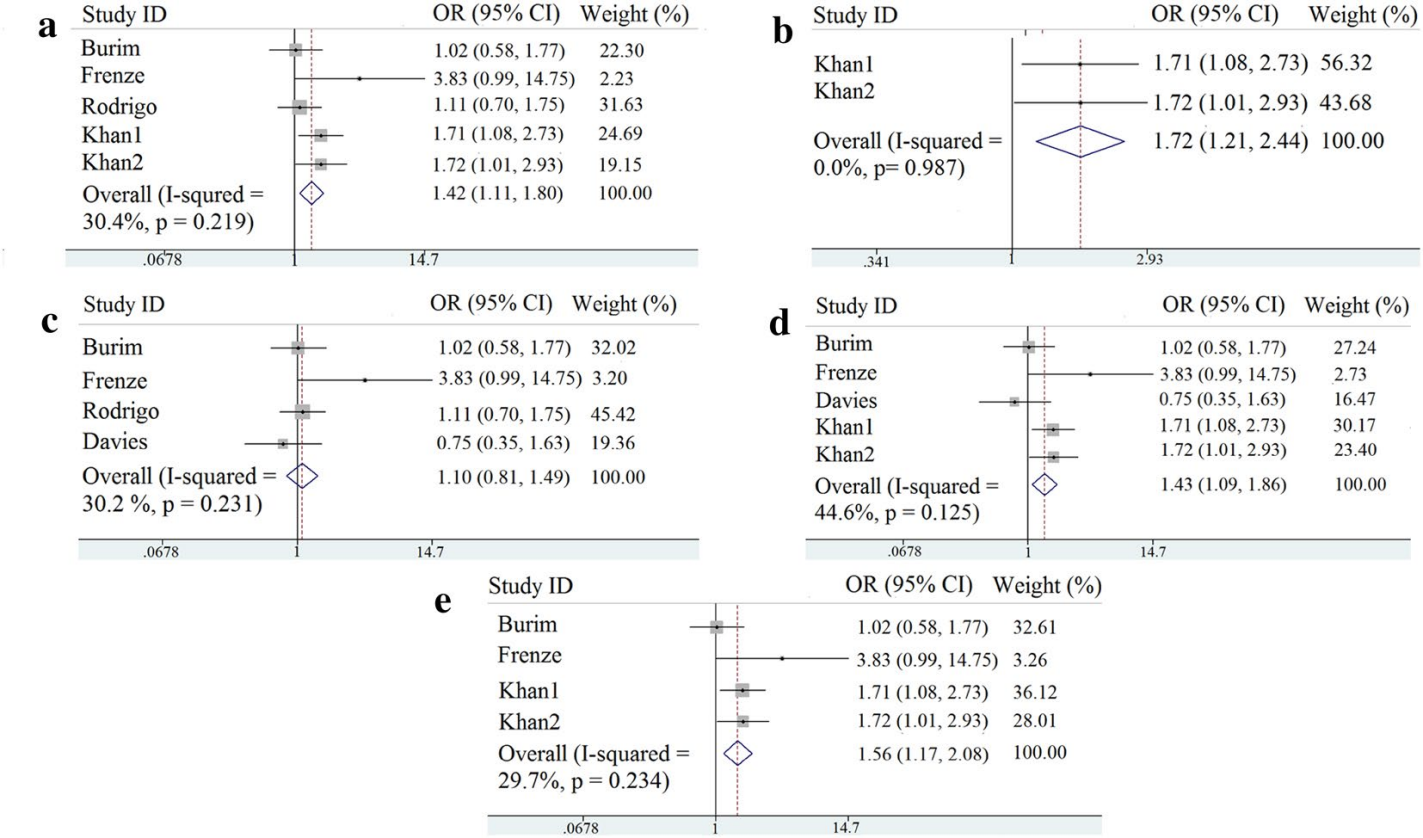
**a** Forest plots of GSTM1 comparison (null vs. active) in articles on alcoholic hepatic cirrhosis; **b** Forest plots of GSTM1 comparison (null vs. active) in Asian population; **c** Forest plots of GSTM1 comparison (null vs. active) in non-Asian population; **d** Forest plots of GSTM1 comparison (null vs. active) in the subgroup of hospital-based studies; **e** Forest plots of GSTM1 comparison (null vs. active) in multiple PCR genotyping method

Classification of diseases, source of population, control source and genotyping method were regarded as the influence factors of the disease. Consequently, subgroup analysis was performed based on cirrhosis type, ethnicity, controlled source and genotyping method, respectively. The analysis results of these subgroups are shown in Table 3. The pooled OR of GSTM1 null in alcoholic hepatic cirrhosis is 1.416 (95% CI 1.112–1.804, *p* = 0.005, Fig. 3a), suggesting that GSTM1 null is a risk factor for alcoholic hepatic cirrhosis. Meanwhile, Asian population with GSTM1 null had significantly increased risks for HC (OR = 1.719, 95% CI 1.212–2.438, *p* = 0.002, Fig. 3b), but this phenomenon was not observed in non-Asians (OR = 1.097, 95% CI 0.806–1.493, *p* = 0.556, Fig. 3c). As to the control source subgroup analysis, GSTM1 null is a risk factor of hepatic cirrhosis in hospital-based studies (OR = 1.426, 95% CI 1.092–1.863, *p* = 0.009, Fig. 3d). The analysis using the multiple PCR genotyping method showed a significant association between GSTM1 null genotype and hepatic cirrhosis occurrence (OR = 1.559, 95% CI 1.171–2.076, *p* = 0.002, Fig. 3e).

**Table 3:** Subgroup analysis of the association between GSTM1 polymorphism and non-viral HC-based on cirrhosis type, ethnicity, controlled source and genotyping method

Subgroup	*N*	Test for association		Test for heterogeneity	
		OR (95%CI)	*p*	*I*^2^ (%)	*p*
Cirrhosis type					
Alcoholic HC	5	1.416 (1.112–1.804)	0.005	30.4	0.219
Ethnics					
Asian	2	1.719 (1.212–2.438)	0.002	0.0	0.987
Non-Asian	4	1.097 (0.806–1.493)	0.556	30.2	0.231
Control source					
HB	5	1.426 (1.092–1.863)	0.009	44.6	0.125
Genotyping method					
Multiple PCR	4	1.559 (1.171–2.076)	0.002	29.7	0.234

Analysis model: fixed effect; *N*: number of eligible group of studies

### Publication bias

**Fig. 4:**
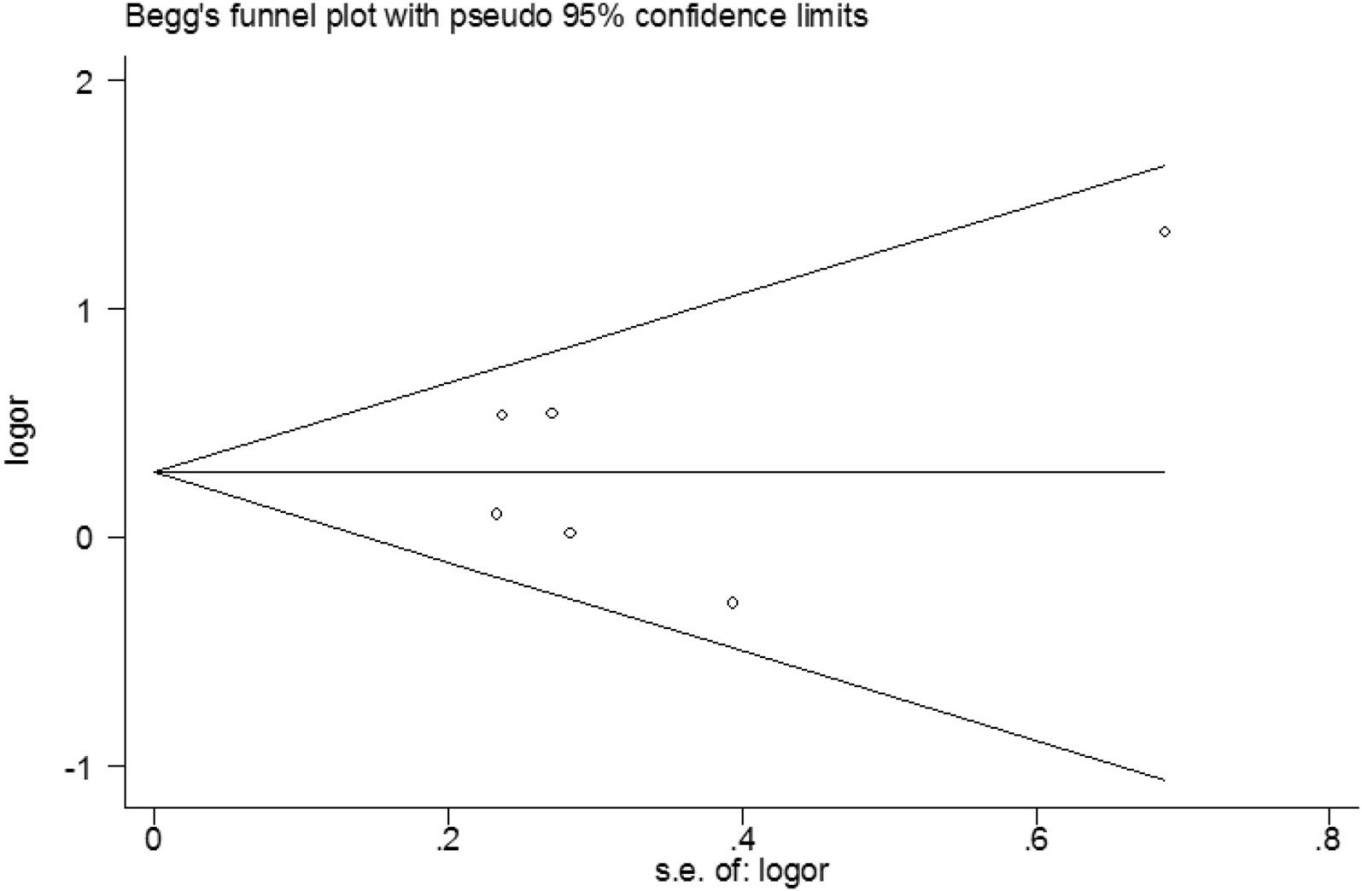
Begg’s test for publication bias of GSTM1 polymorphism

The result of Begg’s test showed that there was no obvious evidence of publication bias (*p* = 0.851). The shape of Begg’s funnel plot is shown in Fig. 4. According to the test results, there was no publication bias in this meta-analysis.

### Sensitivity analysis

**Fig. 5:**
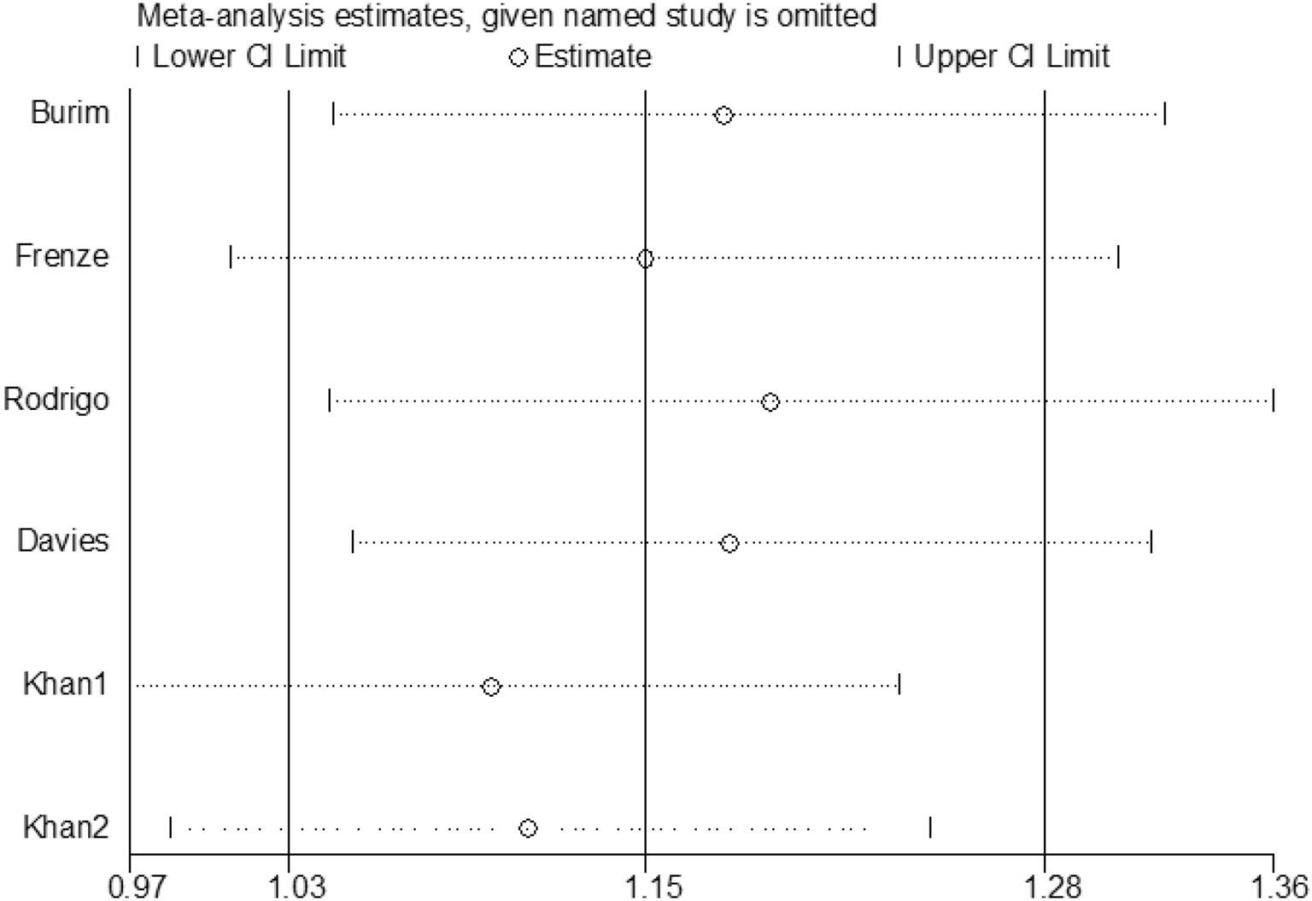
Sensitivity analysis to evaluate the impact of each individual study

To evaluate the influence of each study on the pooled OR, sensitivity analysis was performed and the STATA command “metaninf” is used. The new combined ORs were compared with the original pooled ORs after that one study is expurgated from all eligible articles each time. The results had no significant differences (Fig. 5).

## Discussion

To date, it is the first time to perform meta-analysis to reveal the association between GSTM1 polymorphism and non-viral HC susceptibility. Hepatic cirrhosis is affected by many factors. Among these risk factors, genetic factors have become a research focus now. Many studies showed that GSTs played a crucial role in the etiology of HC. GSTM1 is a common type of GSTs gene. Some researchers reported that GSTM1 polymorphism was associated with the increased risk of non-viral HC. However, other investigations suggested that there was no relationship between GSTM1 polymorphism and non-viral HC. After the combination of these data, results of this meta-analysis revealed that GSTM1 null was a risk factor for susceptibility of HC (OR = 1.337, *p* = 0.013). In addition, results from subgroup analysis classified by ethnicity indicated that HC risk of Asians with GSTM1 null was increased in (OR = 1.719, *p* = 0.002). But it was not applicable for non-Asians. Furthermore, subgroup analysis was also conducted on cirrhosis type. In alcoholic hepatic cirrhosis group, the results are consistent with that of all enrolled studies (OR = 1.416, *p* = 0.005).

Based on the current literature, GSTM1 null is significantly associated with non-viral hepatic cirrhosis risk in Asian population. Interestingly, it was reported that Asian population were more vulnerable to viral hepatic cirrhosis [29]. The popularity of GSTM1 null has been reported to vary with different ethnic populations, 30% of Caucasians while 70% of Asians [14]. The high prevalence of GSTM1 null among Asians may lead to the increased vulnerability to HC. Except for the genetic factors, other factors such as economic and social-cultural factors can also contribute to the development of hepatic cirrhosis. Participants in Khan’s two enrolled studies are Indians. Patients in this region sometimes were reluctant to see the doctor because of economy stresses or lack of education, which interferes the early discovery and treatment of the cirrhosis [30, 31]. This phenomenon can also be observed in the undeveloped area of China, the world’s first ranked incidence and mortality area of HC [32]. It is worthy to notice that the interaction between genetic factors and non-genetic factors may impact the occurrence of non-viral cirrhosis.

Another particular finding of our study was that GSTM1 null could be a significant risk factor for susceptibility of alcoholic HC. Heavy alcohol consumption can promote the formation of reactive oxygen species (ROS) and acetaldehyde, which are both associated with the developing of cirrhosis [33]. However, individual susceptibility to alcoholic cirrhosis varies. Song et al. [34] reported that only approximately 30% of the heavy alcohol consumers developed to liver cirrhosis, suggesting that genetic factors play an important role. GSTM1 activity was involved in the metabolism of xenobiotics and facilitated to protect the cellular from oxidative reactions [20]. Therefore, individuals with GSTM1 null are more likely found to be in risk of hepatic cell damage triggered by excessive alcohol consumption.

This meta-analysis is rigorous. First, this paper is focused on GSTM1 polymorphism and the risk of non-viral hepatic cirrhosis. Studies were selected from three open classic biomedical databases, Pubmed, Web of Science and Embase database. A thorough search strategy was designed. Language type and the period covered by the publications were also limited strictly. Second, objective quality evaluation, particular inclusion criteria and strict exclusion criteria were established to ensure the reliability of this meta-analysis. Finally, the results were generated through appropriate statistics. Sensitivity analysis and stratification analysis were also performed to control the confounding factors.

However, this meta-analysis has limitations to some extent. The data of this meta-analysis were collected from published literature and it is impossible to eliminate publication bias completely. We can only minimize the effect of publication bias to obtain more reliable results. We studied both alcoholic and non-alcoholic HC in the subgroup analysis. However, most of the studies enrolled participants are the excessive drinker. Thus, it is hard to get an accurate result of the non-alcoholic HC in this work. Further well-designed studies focusing on non-alcoholic population with larger sample sizes and different ethnic population are needed to clarify the present findings.

## Conclusion

According to the analysis results of this study, GSTM1 null is associated with the increased risk of non-viral hepatic cirrhosis. Subgroup analysis of cirrhosis type, population, controlled source and detection method also suggest that GSTM1null is a prominent risk factor of hepatic cirrhosis. Thus, GSTM1 polymorphism is related to the pathologies of non-viral hepatic cirrhosis.
